# Dynamic recrystallization behavior and processing map of the Cu–Cr–Zr–Nd alloy

**DOI:** 10.1186/s40064-016-2317-z

**Published:** 2016-05-20

**Authors:** Yi Zhang, Huili Sun, Alex A. Volinsky, Baohong Tian, Kexing Song, Zhe Chai, Ping Liu, Yong Liu

**Affiliations:** College of Materials Science and Engineering, Henan University of Science and Technology, Luoyang, 471003 China; Department of Mechanical Engineering, University of South Florida, Tampa, FL 33620 USA; College of Materials Science and Engineering, University of Shanghai for Science and Technology, Shanghai, 200093 China; Collaborative Innovation Center of Nonferrous Metals, Luoyang, 471003 Henan Province China

**Keywords:** Cu–Cr–Zr–Nd alloy, Hot deformation behavior, Constitutive analysis, Processing map, Microstructure evolution

## Abstract

Hot deformation behavior of the Cu–Cr–Zr–Nd alloy was studied by hot compressive tests in the temperature range of 650–950 °C and the strain rate range of 0.001–10 s^−1^ using Gleeble-1500D thermo-mechanical simulator. The results showed that the flow stress is strongly dependent on the deformation temperature and the strain rate. With the increase of temperature or the decrease of strain rate, the flow stress significantly decreases. Hot activation energy of the alloy is about 404.84 kJ/mol and the constitutive equation of the alloy based on the hyperbolic-sine equation was established. Based on the dynamic material model, the processing map was established to optimize the deformation parameters. The optimal processing parameters for the Cu–Cr–Zr–Nd alloy hot working are in the temperature range of 900–950 °C and strain rate range of 0.1–1 s^−1^. A full dynamic recrystallization structure with fine and homogeneous grain size can be obtained at optimal processing conditions. The microstructure of specimens deformed at different conditions was analyzed and connected with the processing map. The surface fracture was observed to identify instability conditions.

## Background

Cu–Cr–Zr alloy has been considered as a potential material candidate for the railway contact wires, connectors and lead frame materials due to its excellent properties, such as high strength, outstanding electrical conductivity, thermal conductivity, excellent fatigue resistance and formability (Su et al. 2005; Xia et al. 2012; Lin et al. 2011; Bi et al. 2013). In recent years, many investigations have been conducted to improve the deformation characteristics of the Cu–Cr–Zr alloy. However, the hot workability of the Cu–Cr–Zr alloy has been limited, according to previous research (Ding et al. 2013; Ji et al. 2015; Zhang et al. 2015a; Shukla et al. 2015). Thus, it is important to study the hot deformation behavior of the Cu–Cr–Zr alloy. The processing maps corresponding to microstructure have been considered to be the most effective way for this investigation.

According to the dynamic material model (DMM), the processing map developed by Prasad and Seshacharyulu (1998) has been widely used to optimize the hot workability of different types of alloys. Based on the DMM, hot deformation of the work piece can be considered as a process of power dissipation. The total absorbed power (*P*) can be separated into two complementary parts: temperature rising (*G* part) and microstructure evolution (*J* part), with the following mathematical definition:

1 $$P = J + G = \sigma \dot{\varepsilon } = \int\limits_{0}^{\sigma } {\dot{\varepsilon }d\sigma + \int\limits_{0}^{{\dot{\varepsilon }}} {\sigma d\dot{\varepsilon }} }$$

In this model, *G* and *J* can be determined by the parameter *m*, where *m* represents the strain rate sensitivity coefficient of the material. For an ideal linear dissipation process, *m* = 1 and *J* = *J*_max_ = $\sigma \dot{\varepsilon }/2$ = *P*/2, and the value of *J* can be obtained from a dimensionless parameter called the efficiency of power dissipation:

2 $$\eta = \frac{J}{{J_{\hbox{max} } }} = \frac{2m}{m + 1}$$

The power dissipation map was constructed from *η* varied with temperature and strain rate. The *η* value also represents the specific microstructure formation mechanism. Thus, the instability map with a continuum criterion was developed based on the principle of maximum rate of entropy production. It can be expressed as (Xi et al. 2015):

3 $$\xi (\dot{\varepsilon }) = \frac{{\partial \ln \left( {\frac{m}{m + 1}} \right)}}{{\partial \ln \dot{\varepsilon }}} + m < 0$$

The negative $\xi (\dot{\varepsilon })$ represents the flow instability. The variation of $\xi (\dot{\varepsilon })$ with temperature and strain rate constitutes an instability map. The instability map can be superimposed on the power dissipation map, and then the processing map can be constructed. The processing map is important for optimizing processing parameters during the hot working process.

In this study, the flow behavior, dynamic recrystallization behavior and processing maps of the Cu–Cr–Zr–Nd alloys were investigated. The constitutive equations and hot deformation activation energy were developed for the alloy. The critical conditions for dynamic recrystallization (DRX) of the alloy were determined. Based on the dynamic material modeling (DDM), the processing map was constructed to optimize processing parameters and microstructure evolution was observed to validate the processing map.

## Experimental details

The chemical composition (wt%) of the alloy in this study is as follows: 0.8 Cr, 0.3 Zr, 0.05 Nd and Cu balance. The studied alloy was melted in a vacuum induction furnace in argon. The cast ingot with 83 mm diameter and 150 mm length was homogenized at 930 °C for 2 h. Subsequently, the ingot was forged into 25 mm diameter bars. Finally, the forged bars were solution-treated at 900 °C for 1 h followed by immediate water quenching.

The hot compression specimens were cut into cylindrical shape with 8 mm diameter and 12 mm length. The isothermal compression tests were carried out using Gleeble-1500D thermo-mechanical simulator with deformation temperature of 650–950 °C and strain rate of 0.001–10 s^−1^. All the samples were heated to the designed deformation temperature at 5 °C/s heating rate and held for 3 min to make sure the temperature was homogenous throughout the sample. All specimens were compressed and immediately water quenched from the test temperature to maintain deformation microstructure. The deformed specimens were sectioned through longitudinal axis, polished and chemically etched in a solution of FeCl_3_ (5 g) + C_2_H_5_OH (85 ml) + HCl (10 ml). The microstructure was observed using OLYMPUS PMG3 optical microscope and JSM JEOL-5610LV scanning electron microscope. Transmission electron microscopy (TEM) samples were prepared using Gatan 691 ion beam thinner. The JEM-2100 (Jeol, Japan) high resolution transmission electron microscope (HRTEM) was used to analyze the deformation microstructure.

## Results and discussion

### Flow stress behavior

**Fig. 1:**
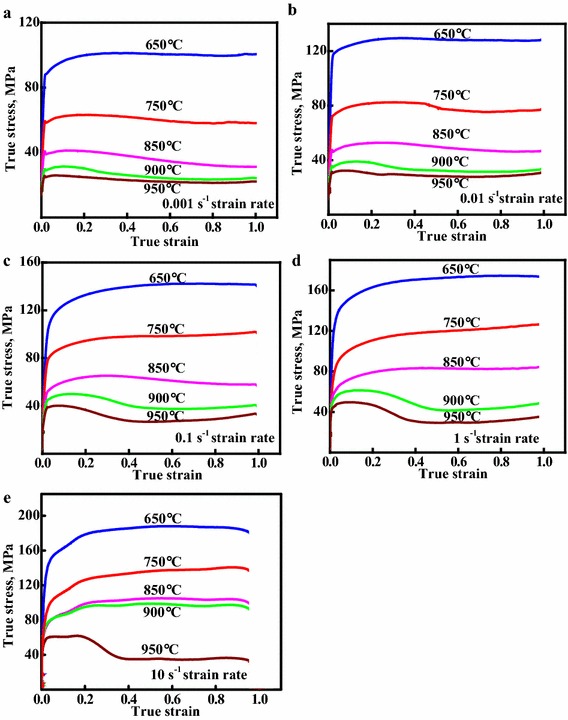
True stress–strain curves of the Cu–Cr–Zr–Nd alloy deformed at: **a** $\dot{\varepsilon }$ = 0.001 s^−1^; **b** $\dot{\varepsilon }$ = 0.01 s^−1^; **c** $\dot{\varepsilon }$ = 0.1 s^−1^; **d** $\dot{\varepsilon }$ = 1 s^−1^ and **e** $\dot{\varepsilon }$ = 10 s^−1^

**Fig. 2:**
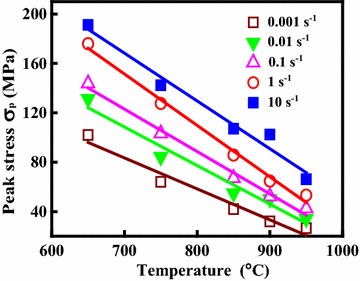
Peak stress of the true stress–strain curves for the Cu–Cr–Zr–Nd alloy under different deformation conditions

The true stress–true strain curves of the Cu–Cr–Zr–Nd alloy obtained at the strain rate varied from 0.001 to 10 s^−1^ and the deformation temperature varied from 650 to 950 °C are shown in Fig. 1. It can be seen that the flow stress strongly depends on the deformation temperature and the strain rate. The flow stress increases with the strain rates at constant temperature. The reason for this is because the dynamic recovery (DRV) and dynamic recrystallization (DRX) have enough time to complete at low strain rate, so the effect of work hardening can be offset by the softening effect. According to Fig. 1, at the initial deformation stage, there is an obvious work hardening stage observed. The work hardening in the initial stage is associated with the increment of dislocation density, which can effectively hinder dislocation movement (Zhang et al. 2015b). After that, the flow stress increases to a maximum and then decreases to a steady state value for the alloy deformed at 900 and 950 °C, respectively. This phenomenon is characteristic for hot working accompanied by dynamic recrystallization (Galiyev et al. 2001). However, typical continuous strain hardening was observed at the deformation temperature of 650 °C in Fig. 1. This is because the effect of work hardening is stronger than the effect of dynamic softening. As illustrated earlier, the strain hardening and strain softening can be effectively controlled by changing dislocations movement (Abbasi and Shokuhfar 2007). Variations of the peak stress (*σ*_p_) with temperature and strain rate are shown in Fig. 2. The peak stress increases with the increase of strain rate and the decrease of deformation temperature.

**Fig. 3:**
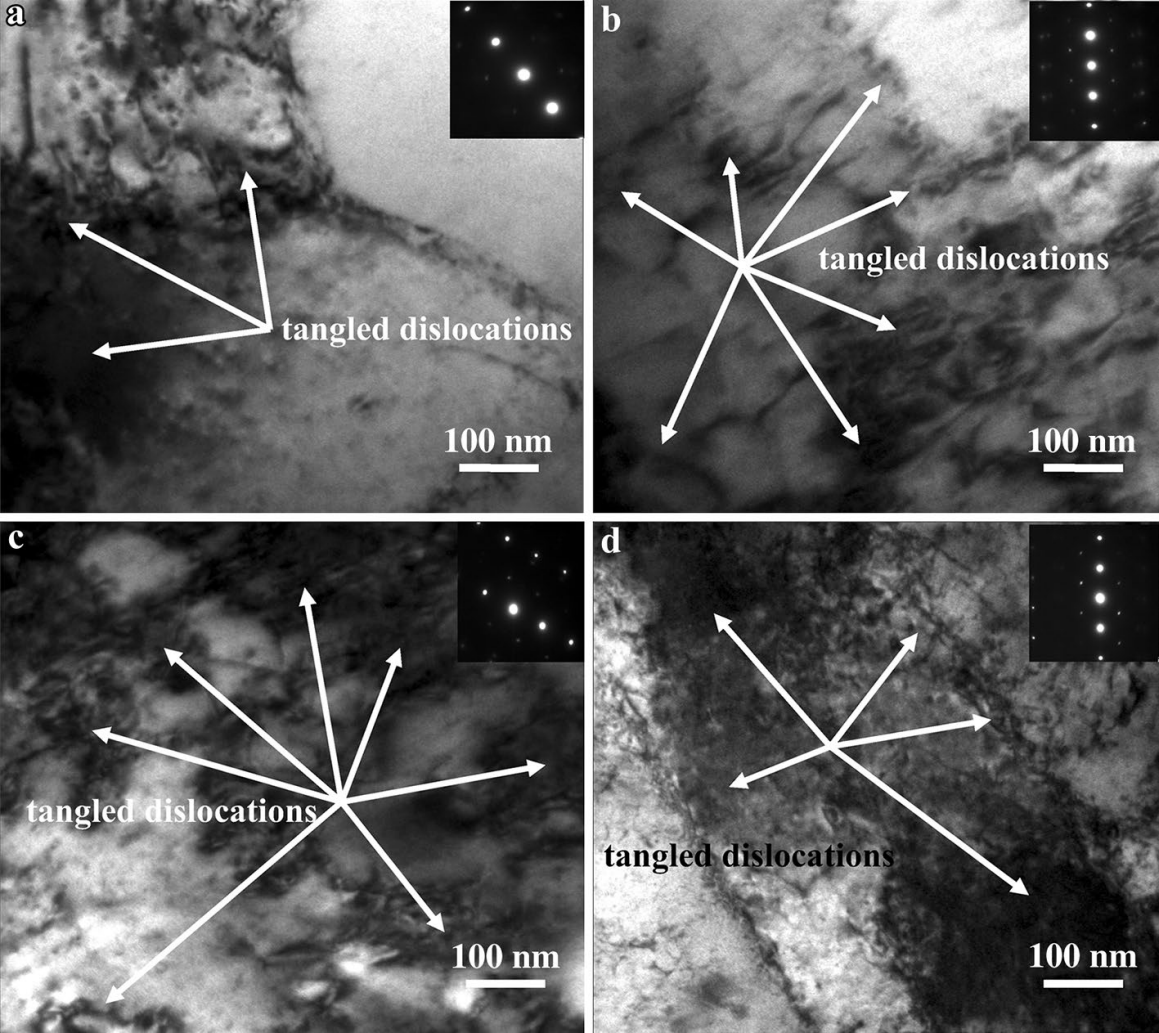
TEM micrographs of the Cu–Cr–Zr–Nd alloy deformed at strain of 0.7 and different deformation conditions: **a** 950 °C, 0.001 s^−1^; **b** 850 °C, 0.01 s^−1^; **c** 750 °C, 1 s^−1^ and **d** 650 °C, 10 s^−1^

Figure 3 shows TEM micrographs of the Cu–Cr–Zr–Nd alloy deformed at the strain of 0.6 and different deformation conditions. Figure 3a shows the TEM micrographs of the Cu–Cr–Zr–Nd alloy deformed at 950 °C and the strain rate of 0.001 s^−1^. It can be seen that the density of dislocations is relatively low under this condition. Figure 3b shows that the dislocations marked by arrows are tangled and stored in the grain interior of the Cu–Cr–Zr–Nd alloy deformed at 850 °C with the 0.01 s^−1^ strain rate. Figure 3c shows the dislocation pile-up deformed at 750 °C and the strain rate of 1 s^−1^. The density of dislocations is much higher than in Fig. 3b. Therefore, the corresponding flow stress in Fig. 3c is much higher than in Fig. 3b. The increase in the number of dislocations is observed in Fig. 3d. Compared with Fig. 3c, dislocations pile up more and the dislocation density is much higher. According to the calculations from the SAED patterns, the interplanar spacing in Fig. 3d is much higher than in Fig. 3c. This also indicates that the dislocation density is much higher in Fig. 3d. Thus, the high density dislocations are intersected and tangled, forming the network structure, which makes dislocation slip more difficult (Ning et al. 2011). It can be found that the flow stress is about 120 MPa, corresponding with Fig. 3c, and the flow stress is close to 200 MPa under the deformation conditions of Fig. 3d.

### Activation energy and constitutive equations

During the hot deformation, the relationship between the flow stress, strain rate and deformation temperature can be represented by the Arrhenius equation expressed as (Pu et al. 2014; Sellars and McTegart 1966; Zener and Hollomon 1944):

4 $$\dot{\varepsilon }\exp (Q/RT) = A_{1} \sigma^{{n_{1} }}$$

5 $$\dot{\varepsilon }\exp (Q/RT) = A_{2} \exp (\beta \sigma )$$

6 $$\dot{\varepsilon }\exp (Q/RT) = A[\sinh (\alpha \sigma )]^{n}$$

Here, $\dot{\varepsilon }$ is the strain rate (s^−1^), *T* is the absolute temperature (K), *Q* is the activation energy of DRX (kJ/mol), *σ* is the flow stress (MPa) for a given stain, *R* is the universal gas constant (8.314 J/mol K), *A* (s^−1^), *A*_1_, *A*_2_, *n*_1_, *n* and *α* (MPa^−1^) are the materials constants (*α* = *β*/*n*_1_). Taking natural logarithms of both sides of Eqs. (4) and (5) yields:

7 $$\ln \dot{\varepsilon } = \ln A_{1} + n_{1} \ln \sigma - Q/RT$$

8 $$\ln \dot{\varepsilon } = \ln A_{2} + \beta \sigma - Q/RT$$

**Fig. 4:**
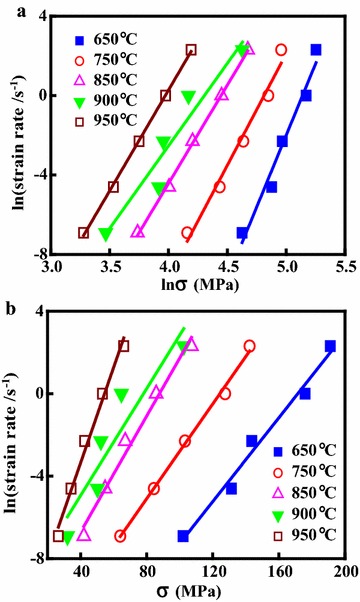
Relationships between: **a** ln$\dot{\varepsilon }$ and ln*σ*; **b** ln$\dot{\varepsilon }$ and *σ*

A linear relationship exists between ln$\dot{\varepsilon }$ and ln*σ* with linear slope *n*_1_, shown in Fig. 4a. A linear relationship exists between ln$\dot{\varepsilon }$ and *σ* with the linear slope *β* shown in Fig. 4b. The values of *n*_1_ and *β* can be calculated from the average values of the slopes: *n*_1_ = 7.193, *β* = 0.101. Thus, the *α* value of the alloy is calculated as *α* = *β*/*n*_1_ = 0.014 MPa^−1^. Taking natural logarithms of both sides of Eq. (6) yields:

9 $$\ln \left[ {\sinh \left( {\alpha \sigma } \right)} \right] = - \ln A/n + \ln \dot{\varepsilon }/n + Q/nRT$$

Taking partial derivatives of Eq. (9) into consideration yields:

10 $$Q = R\left[ {\frac{{\partial (\ln \dot{\varepsilon })}}{\partial \ln [\sinh (\alpha \sigma )]}} \right]_{T} \left[ {\frac{\partial \ln [\sinh (\alpha \sigma )]}{\partial (1/T)}} \right]_{{\dot{\varepsilon }}} = RnS$$

**Fig. 5:**
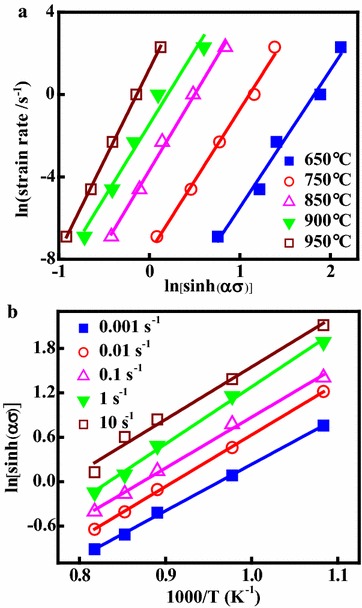
Relationship between: **a** ln[sinh(*ασ*)] and ln$\dot{\varepsilon }$; **b** ln[sinh(*ασ*)] and 1000/T

The relationships of ln$\dot{\varepsilon }$–ln[sinh(*ασ*)] and ln[sinh(*ασ*)] − 1/*T* at different temperatures are shown in Fig. 5a, b, respectively. As a result, the values of *n* and *S* can be calculated by means of linear regression analysis. The value of *Q* during hot compression can be obtained as *Q* = *RnS* = 404.84 kJ/mol.

**Fig. 6:**
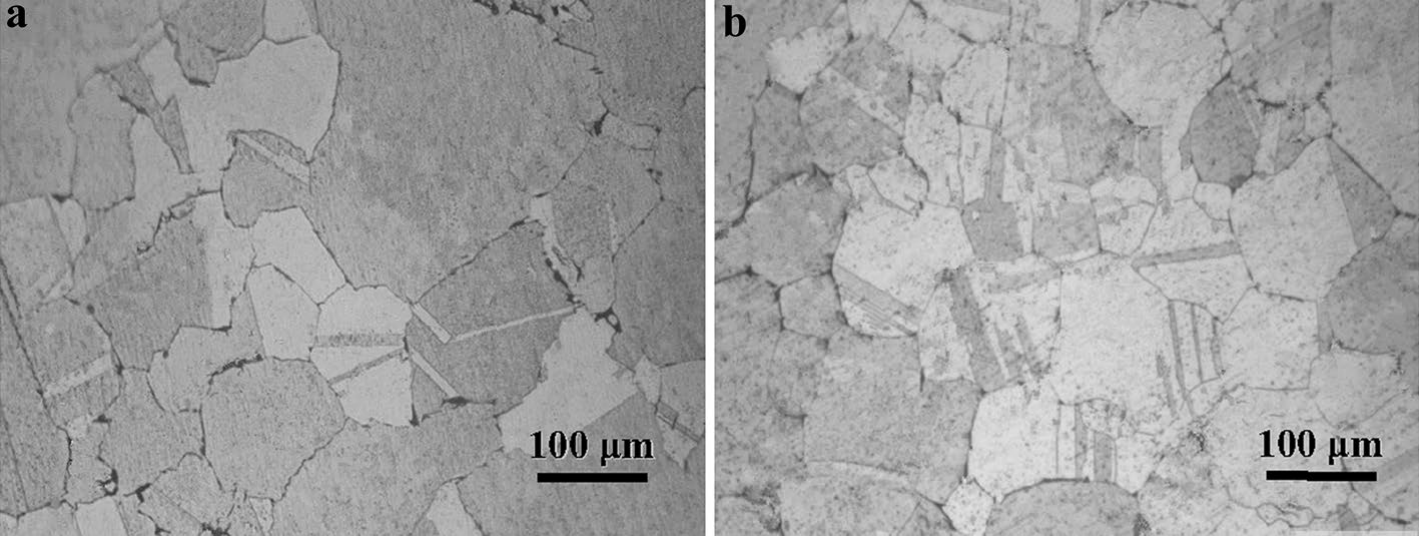
Microstructure of **a** Cu–Cr–Zr and **b** Cu–Cr–Zr–Nd alloys after solution treatment at 900 °C for 1 h

The activation energy as an indicator of the degree of difficulty of plastic deformation is an important physical parameter (Liao et al. 2015). The *Q* value of this alloy is a little lower than that obtained at similar deformation conditions of the Cu–0.36Cr–0.03Zr alloy (432.6 kJ/Mol) and the Cu–0.6Cr–0.03Zr alloy (572.053 kJ/mol), compared with references Ding et al. (2013) and Ji et al. (2015), respectively. The higher activation energy *Q*, the harder the plastic deformation. This indicates that dislocation movement and DRX are easier to occur for this experimental alloy. It also means that this experimental alloy has good hot workability. Compared with the above references, the addition of Nd can refine the grains of the Cu–Cr–Zr alloy, shown in Fig. 6a, b, respectively. According to the statistical measurements, the mean grain sizes of the Cu–Cr–Zr and Cu–Cr–Zr–Nd alloys were approximately 69 and 60 μm, respectively. Thus, the addition of Nd can refine the grain and improve dynamic recrystalization during hot deformation. The reason is that DRX nucleation is improved by the increase of the boundaries area. Chen et al. (2007) found that the boundary movement and grain rotation can be promoted because of the fine recrystallized grains during hot deformation.

Over a wide range of temperatures and strain rates, the Arrhenius equation also can be written as (Kil et al. 2015; Wang et al. 2015a; Etaati and Dehghani 2013; Morakabati et al. 2011; Spigarelli et al. 2003):

11 $$Z = \dot{\varepsilon }\exp (Q/RT) = A[\sinh (\alpha \sigma )]^{n}$$

Taking natural logarithms of both sides of Eq. (11) yields:

12 $$\ln Z = \ln A + n\ln [\sinh (\alpha \sigma )]$$

**Fig. 7:**
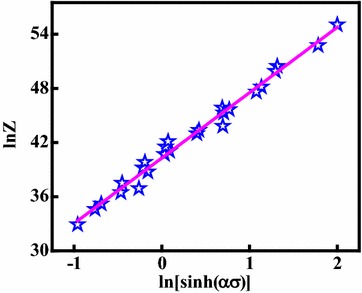
The Zener–Hollomon parameter, *Z*, as a function of the flow stress

The relationship between the ln[sinh(*ασ*)] and ln*Z* is shown in Fig. 7. It can be found that the correlation coefficient for the linear regression is 0.986, which demonstrates the accuracy of Eq. (12) for describing hot deformation behavior of the alloy. The ln*A* is the intercept of the ln[sinh(*ασ*)] and ln*Z* plot, so the value of *A* is 3.114 × 10^17^. Thus, the constitutive equation of the Cu–Cr–Zr–Nd alloy can be confirmed as:

13 $$\dot{\varepsilon } = 3.114 \times 10^{17} [\sinh (0.014\sigma )]^{7.193} \exp ( - 404.84/RT)$$

### Processing maps

**Fig. 8:**
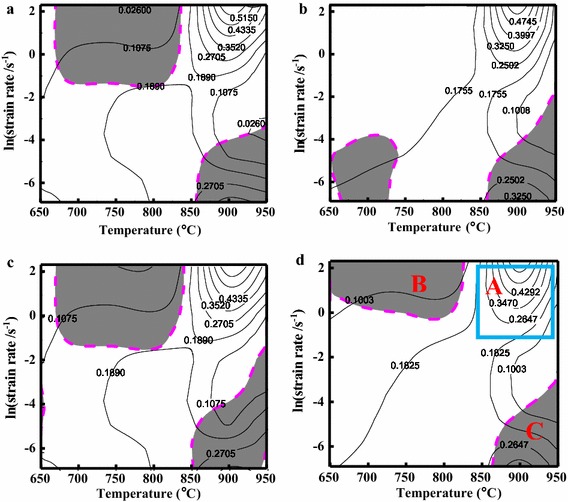
Processing maps for the Cu–Cr–Zr–Nd alloy at a true strain of: **a** 0.3; **b** 0.4; **c** 0.5 and **d** 0.6

According to the above analysis and the flow stress data obtained in the isothermal compression tests, the processing maps of the Cu–Cr–Zr–Nd alloy deformed at the strain of 0.3, 0.4, 0.5 and 0.6 are shown in Fig. 8a–d, respectively. The contours represent the efficiency of power dissipation, and the shaded regions indicate the flow instability domains (the values of $\xi (\dot{\varepsilon })$ are negative). It can be seen that the peak efficiency of power dissipation (*η*) was slightly impacted by the strain. A domain with about 45 % *η* and the location of the domain is similar in all figures. Many researchers have indicate that the high value of *η* means that the material dissipates more energy through microstructural changes, such as DRX, which is considered to be the best deformation mechanism and provides relatively stable flow stress and produces fewer defects (Karami and Mahmudi 2012; Evans and Scharning 2001; Bhattacharya et al. 2012). However, it should be noted that the regimes with high *η* may represent unstable flow, usually manifested in the form of cracks and/or deformation bands (Prasad and Rao 2005).

From Fig. 8a, c, there are two similar observed shaded domains. One is at the deformation temperature range of 650–820 °C and the strain rate range of 0.1–10 s^−1^. The other one is at the deformation temperature range of 850–950 °C and the strain rate range of 0.001–0.01 s^−1^, which was also obtained in Fig. 8b, d. At the true strain of 0.4 in Fig. 8b, another shaded domain was observed at the deformation temperature range of 650–720 °C and the strain rate range of 0.001–0.01 s^−1^. Similar research results have been reported where flow instability occurred at lower temperatures and higher strain rates because of the possible presence of shear bands and cracks (Mahmudi et al. 2011; Kim et al. 2012; Li et al. 2011).

At the strain of 0.6, Domain A has a stable region with a peak efficiency of power dissipation shown in Fig. 8d. It revealed a greater efficiency of power dissipation in the temperature range of 900–950 °C and the strain rate range of 0.1–1 s^−1^, with a peak efficiency of about 43 %. Many researchers have indicated that the value of *η* corresponding to DRX is about 30–50 % (Lin et al. 2008). Thus, this domain is considered as the safe region for hot deformation. The second region is represented as the domain B with negative values for the instability parameter. Domain B occurs in the temperature range of 650–820 °C and the strain rate range of 1–10 s^−1^. At the high strain rate, deformation time is too short for dissipating generated heat, and the temperature increases with localized plastic flow leading to shear bands and cracks in the shear planes formation (Ning et al. 2012; Sarebanzadeh et al. 2011). Therefore, this instability region should be avoided during hot deformation. Domain C occurs in the temperature range of 850–950 °C and the strain rate range of 0.001–0.01 s^−1^. In this instability region, the dynamic recrystallization grains become coarsened at high temperature and low strain rate. Thus, hot working in these regions is also considered to be unsafe. According to the processing maps of the Cu–Cr–Zr–Nd alloy, the optimal hot working conditions are in the temperature range of 900–950 °C and the strain rate range of 0.1–1 s^−1^.

### Microstructure evolution

Many research results indicated that the DRV and DRX were the main softening mechanisms during the hot deformation processes (Cheng et al. 2014; Dong et al. 2006; Sharma et al. 2005; Kai et al. 2015). The DRX can reduce the rate of work hardening and promote the hot workability of alloys (Kai et al. 2015; Srinivasan and Prasad 1994; Yin et al. 2013). Thus, different domains in the processing maps can be interpreted according to the microstructure evolution during DRV, DRX and super-plastic deformation. These are considered as beneficial mechanisms, while voids and cracks are harmful mechanisms (Dobatkin et al. 2015; Ning et al. 2010; Wang et al. 2013; Liu et al. 2014).

**Fig. 9:**
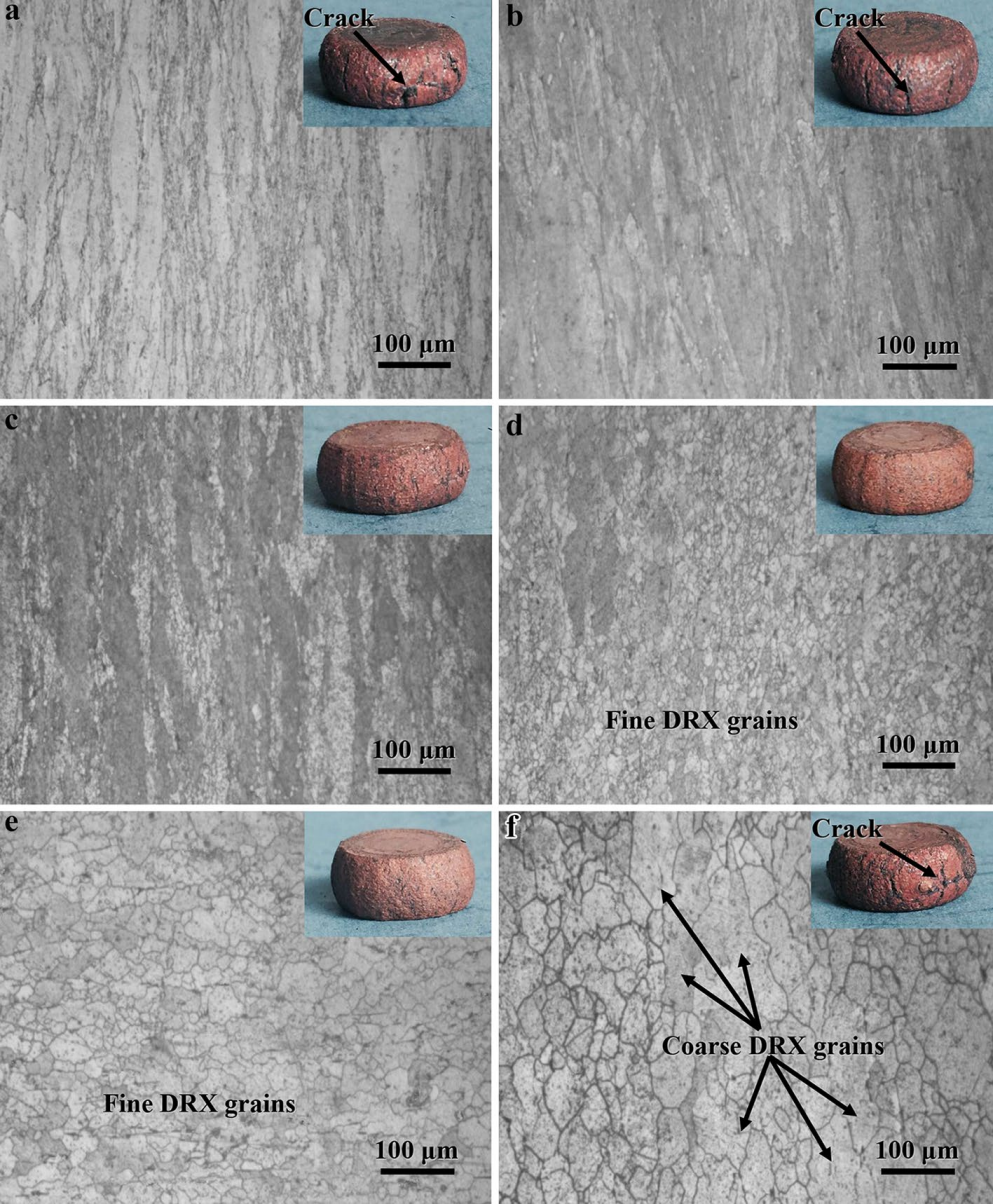
Optical images of the microstructure of the hot compressed specimens deformed at strain of 0.6 and different conditions: **a** 650 °C, 1 s^−1^; **b** 750 °C, 1 s^−1^; **c** 850 °C, 0.1 s^−1^; **d** 900 °C, 1 s^−1^; **e** 950 °C, 0.1 s^−1^ and **f** 950 °C, 0.001 s^−1^, where the *insets* show the corresponding specimen pictures

Figure 9 shows typical microstructure of the Cu–Cr–Zr–Nd alloy deformed at 0.6 strain and different deformation conditions. It can be seen that the grains were obviously elongated in Fig. 9a at 650 °C and the strain rate of 1 s^−1^. There are no obvious recrystallized grains observed, except for the shear zone. In Fig. 9b, only a few recrystallized grains were observed in the grain boundaries. This means that the main softening mechanism is dynamic recovery in the domain B. The instability mechanisms are associated with cracking, localized plastic flow or adiabatic shear bands at low temperature and high strain rate conditions (Sun et al. 2011). Thus, these hot deformation conditions could trigger the appearance of surface cracks, which could be indicated visually by the surface investigation of the deformed specimens, shown as the inserts in Fig. 9a, b, respectively. The microstructure of the alloy deformed at 850 °C and 0.1 s^−1^ is shown in Fig. 9c. A typical necklace-type structure was observed, which is the main mechanism of recrystallization nucleation (Momeni et al. 2014). Some recrystallized grains around original grain boundaries and some elongated grains are still present in the microstructure. This means that the DRX is incomplete and the microstructure is called mixed-grain microstructure (Wang et al. 2015b). Based on the above results and analysis, these hot deformation conditions should be avoided for this alloy.

With the increasing temperature, the specimen deformed in the domain A exhibits completed DRX structure in Fig. 9d, e, respectively. Some new and fine DRX grains are observed. Both specimens are deformed in the domain A with high efficiency (>0.3), corresponding with Fig. 9d. Shi et al. (2015) reported that the completed DRX in the stability region has high efficiency of power dissipation. Lu et al. (2013) found that the highest efficiency of power dissipation was obtained when the DRX was fully completed. Comparing Fig. 9d, e, the DRX grains deformed at 950 °C and 0.1 s^−1^ obviously grow in size, mainly due to recrystallized grains at high temperature having large driving force for nucleation and growth (Kong et al. 2015). This means that the grain boundary bulging through strain-induced grain boundary migration is the dominant nucleation mechanism of DRX. However, the recrystallized grains are still fine and homogenous. It can be concluded that the above hot deformation conditions represent the optimal processing window. The microstructure of the specimen deformed in the instability region at the strain rate of 0.001 s^−1^ and temperature of 950 °C (domain C) is shown in Fig. 9f. It can be seen that the DRX grains marked by arrow become coarse. Surface cracks were also observed in the insert of Fig. 9f.

**Fig. 10:**
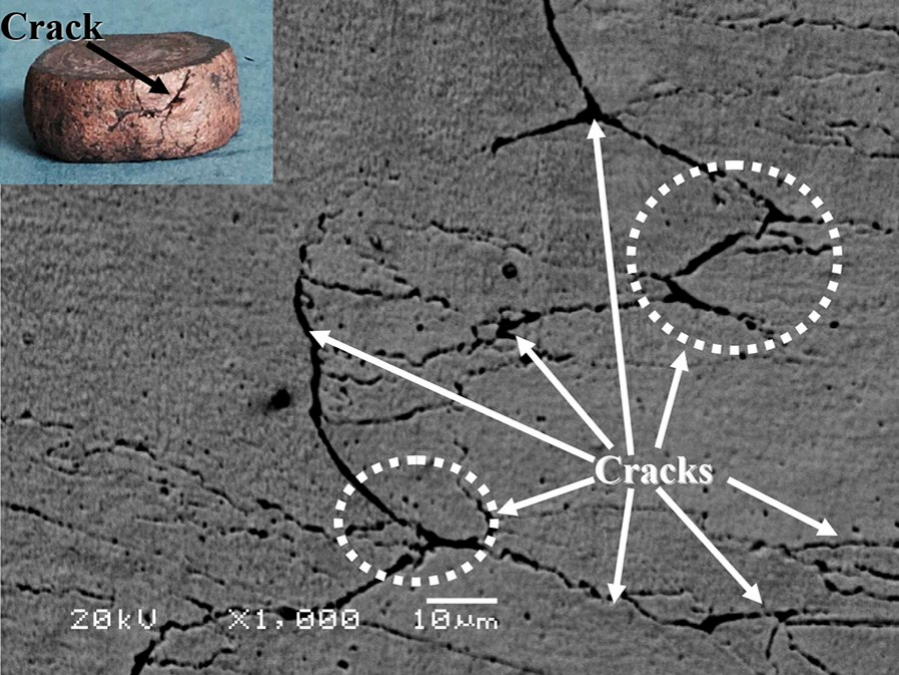
SEM images showing cracks in the compressed specimen at deformation temperature of 650 °C and strain rate of 10 s^−1^, where the *insets* show the corresponding specimen picture

SEM images of the alloy deformed at the strain rate of 10 s^−1^ and 650 °C are shown in Fig. 10. The cracks marked by the arrows appeared in the alloy at these deformation conditions. The instability mechanisms are associated with cracking at low temperature and high strain rate (Lv et al. 2014). This is in a good agreement with the processing maps results. Therefore, the alloy can easily fracture during deformation processing, and this region therefore should be avoided in industrial practice.

## Conclusions

Hot deformation behavior and the microstructure of the Cu–Cr–Zr–Nd alloy have been investigated in the 650–950 °C temperature range and the 0.001–10 s^−1^ strain rate range. The following conclusions can be drawn from this investigation:

1. The flow stress strongly depends on the deformation temperature and the strain rate. The flow stress increases with the strain rate at constant temperature, and decreases with the deformation temperature at a constant strain rate. The flow curves exhibited typical characteristics of dynamic recrystallization at high temperatures and low strain rates.
2. The apparent activation energy for hot deformation of the Cu–Cr–Zr–Nd alloy is 404.84 kJ/mol. The constitutive equation for the flow stress can be expressed as:
  $$\dot{\varepsilon } = 3.114 \times 10^{17} [\sinh (0.014\sigma )]^{7.193} \exp ( - 404.84/RT).$$
3. Based on the DMM principles, the processing maps at the strain of 0.3, 0.4, 0.5 and 0.6 were established. According to the analysis of processing maps data and microstructure observations, the optimal hot working processing parameters for the Cu–Cr–Zr–Nd alloy are in the temperature range of 900–950 °C and the strain rate range of 0.1–1 s^−1^. Full dynamic recrystallization structure with fine and homogeneous grain size can be obtained at optimum conditions.
